# Tablet terminals: a useful tool to explain in vitro fertilization treatment

**DOI:** 10.1186/s40738-017-0031-3

**Published:** 2017-01-23

**Authors:** Atsushi Yanaihara, Shirei Ohgi, Kenichirou Motomura, Takumi Yanaihara

**Affiliations:** Yanaihara Women’s Clinic, 1-26-29 Ofuna, Kamakura, Kanagawa Japan Zip247-0056

**Keywords:** Tablet terminal, Explanation, IVF

## Abstract

**Background:**

Explanations that involve medical care treatment take time. This also applies to explanations of in vitro fertilization (IVF) in the field of infertility treatment. This is because the cause of infertility differs from couple to couple, and because the explanations must begin with the mechanism of pregnancy. Recently, explanations facilitated by tablet terminals have been used in the field of medicine. In the present study, the efficacy and problems of explanations facilitated by tablet terminals were evaluated and compared with the explanations of IVF facilitated by paper-based pamphlets.

**Methods:**

A total of 249 couples were asked to read a paper-based pamphlet explaining IVF treatment, while 252 couples were asked to view an explanation on a tablet terminal. The patients then answered a seven-item questionnaire. The answers to the questionnaire were based on a three-point scale, and statistical analysis was performed with the Mann-Whitney *U* test.

**Results:**

Patients responded that the explanation facilitated by the tablet terminal was significantly easier to understand for all seven questionnaire items (*p* <0.05).

The answer ‘I did not understand’ was selected for the items related to ‘The treatment fees’ (4.8% of answers) and ‘Things to take note of, such as consultation times’ (6.7% of answers).

**Conclusion:**

While patients generally did not understand the mechanism of pregnancy, explanations of IVF treatment facilitated by a tablet terminal were found to be more effective than paper-based explanations, although there is room for improvement.

## Background

The provision of an explanation for patients prior to treatment (and consent for treatment) has become commonplace. Patients often have very little medical knowledge or understanding of the mechanisms needed to achieve pregnancy. The recent ubiquity of the Internet has created an environment in which information is easier to obtain than ever before. Nonetheless, patients may have trouble fully comprehending biological mechanisms and medical procedures.

One-hundred patients who were initial diagnosed and had not been pregnant previously answered a question about the mechanism of pregnancy in our clinic; 85% of the patients answered that they did not understand the mechanism.

It is thus the duty of medical professionals to find ways to increase patients’ understanding so that they may make the best treatment decisions for themselves and their families.

In the case of infertility treatment, changes in the social context have recently led to the development of in vitro fertilization (IVF) as an essential therapy. According to the 2016 Declining Birthrate White Paper in Japan, the average age at marriage was 29.4 years. It has increased by 2.2 years compared with 14 years ago. The average age of a primipara has also increased to 30.1 years. This has been explained by the later age at marriage and the social progress of women. Many patients coming to the hospital in our clinic are 35 years old or older.

While the level of treatment should, in principle, be increased gradually, there are some cases of infertility in which treatment must begin with IVF, which is an advanced therapy. Some facilities provide specialist consultants, and, ideally, explanations of treatment should be given on an individual basis; however, a survey of 60 facilities in Japan found that, of 16 facilities that offered individual explanations, counsellors gave explanations at two facilities, and doctors provided explanations at 14 facilities, while the remaining 44 facilities (73%) provided explanations with joint briefings. At our hospital, we offer individual guidance from a doctor; however, this may take up to 2 h per couple, which is quite inefficient. The incorporation of a system of joint briefings, such as those implemented at 73% of facilities, would be an acceptable alternative. Joint briefings may also be effective in explaining the methodology of IVF to patients. However, Japan has a cultural background in which people do not openly ask questions or express doubt in front of others, which puts into question the adequacy of patients’ understanding of explanations. Moreover, the cause of infertility differs from couple to couple, so it is necessary to explain first why IVF is a required treatment for a particular couple, and then have them understand the context in which they will undergo IVF. This part of the process is impossible to complete with joint briefings. The personal consultation takes about 2.5 h, the same as for joint briefings.

At our hospital, we initially asked patients to prepare themselves with a paper-based pamphlet, and then visit our hospital for an in-person consultation, which takes about 2.5 h. As tablet terminal use has increased, the need to explain how to use them has greatly decreased. We therefore created our own IVF explanation program that takes 30 min to watch, and examined whether tablet terminals can serve as adequate tools for providing explanations to patients. This method has already been used in the study of anatomy and in surgical settings [1, 2]; however, there are no reports of this method related to reproductive medicine.

## Methods

The patients were divided into two groups: 249 couples were asked to read a paper-based pamphlet providing an explanation of IVF treatment, and 252 couples were asked to view an explanation on a tablet terminal. The patients then answered a seven-item questionnaire.

The contents of the explanation were the same. The paper-based pamphlet included a picture and a photograph, and the tablet terminal included a photograph and a video to explain pregnancy mechanisms, the growth process of the egg, and other factors.

Questionnaires were distributed to a total of 501 couples and had a response rate of 100%.

The questionnaire contained the following items, and answers were given based on a three-point scale (‘I understood well’ = 3 points, ‘I understood it’ = 2 points, and ‘I did not understand = 1 point’):

1. The mechanism of spontaneous pregnancy and the causes of infertility
2. The course of IVF treatment
3. The different methods of fertilization (difference between insemination and micro-fertilization)
4. The culture period (difference between day 3 and day 5 embryo transfers)
5. The treatment fees
6. The schedule of IVF treatment
7. Factors to take note of, such as consultation times

Patients were also asked to include any queries in the provided remarks column.

The Mann-Whitney *U* test was used for statistical analysis of the total scores, with a level of significance of *p* <0.05.

Informed consent was obtained from all couples.

## Results

Patients responded that the explanation facilitated by a tablet terminal was significantly easier to understand for all seven questionnaire items (*p* <0.05).

For item (1), 73.4% answered “I understand well”, and 99.2% answered “I understand well + I understand it” with the tablet, compared to 36.9 and 76.0% with the paper-based pamphlet, respectively.

For item (2), the respective percentages were 63.8 and 100% with the tablet terminal, respectively, and 22.8 and 70.2% with the paper-based pamphlet, respectively. For item (3), they were 55.5 and 100% with the tablet terminal, respectively, and 27.7 and 3.7% with the paper-based pamphlet, respectively.

For item (4), they were 54.3 and 96.8% with the tablet terminal, respectively, and 0 and 53.0% with the paper-based pamphlet, respectively. For item (5), they were 43.2 and 95.2% with the tablet terminal, respectively, and 0 and 53.0% with the paper-based pamphlet, respectively.

For item (6), they were 39.6 and 98.4% with the tablet terminal, respectively, and 27.7 and 75.0% with the paper-based pamphlet, respectively. For item (7), they were 42.0 and 93.2% with the tablet terminal, respectively, and 30.5, 61.8% with the paper-based pamphlet, respectively.

For items (4) and (5), 0% of patients answered ‘I understand well’ with the paper-based pamphlet explanation.

In particular, none of the patients answered, ‘I did not understand’ to items (2) or (3) with the tablet terminal explanation.

However, an answer of ‘I did not understand’ was given for items (5) and (7), at rates of 4.8% and 6.7%, respectively, for the tablet terminal explanation (Table 1).

**Table 1:** The questionnaires results

	I did not understand it	I understood it	I understood well	
(1) The mechanism of spontaneous pregnancy and the causes of infertility				
paper-based pamphlet(*n* = 249)	59	98	92	
tablet terminal(*n* = 252)	2	65	185	*P* <2.2e-16
(2) The course of IVF treatment				
paper-based pamphlet(*n* = 249)	74	118	57	
tablet terminal(*n* = 252)	0	91	161	*P* <2.2e-16
(3) The different methods of fertilization (difference of insemination and micro-fertilization)				
paper-based pamphlet(*n* = 249)	66	114	69	
tablet terminal(*n* = 252)	0	112	140	*P* <2.2e-16
(4) The culture period (difference of day3 and day5 embryo transfer)				
paper-based pamphlet(*n* = 249)	117	132	0	
tablet terminal(*n* = 252)	8	107	137	*P* <2.2e-16
(5) The treatment fees				
paper-based pamphlet(*n* = 249)	117	132	0	
tablet terminal(*n* = 252)	12	131	109	*P* <2.2e-16
(6) The schedule of IVF treatment				
paper-based pamphlet (*n* = 249)	62	118	69	
tablet terminal(*n* = 252)	4	148	100	*P* = 2.678e-8
(7) Things to take note of, such as consultation times				
paper-based pamphlet(*n* = 249)	95	78	76	
tablet terminal(*n* = 252)	17	129	106	*P* = 1.849e-9

Queries in the remarks column included questions about stimulation methods (long, short, mild, etc.) from 26 patients, and assisted hatching (timing, method, etc.) from 25 patients (Table 2).

**Table 2:** The results of remarks column

Questions	number
About ovarian stimulation method	26
About assisted hatching	25
About day of transfer (day3 or day5)	15
The side effect of the medicine	11
The treatment fees	11
About being careful in everyday life	8
About anesthesia	7

## Discussion

Although we are able to obtain information easily with the ubiquity of the Internet, patients cannot make sense of the information. Correct information and wrong information are often mixed, and this is the cause of their confusion.

In the present study, sufficient essential information was provided to the patients, so that the patients’ knowledge could facilitate their treatment without incident. However, time for consultations is short in the clinical setting, and it is therefore beneficial to have a tool that facilitates explanations and understanding.

The tablet terminal is ubiquitous in the medical field, and its use to facilitate patient knowledge has begun [1–3]. Although terminal tablets are expected to become a widely-used tool in the field of reproductive medicine, it is necessary for us to know the limits and strengths of tablet terminal tools.

Thus, explanations of IVF treatment provided using a paper-based pamphlet and using a tablet terminal were compared, and patients’ understanding of the information with these methods was evaluated using a questionnaire.

Answers that included ‘I did not understand’ were given for (1) ‘The mechanism of spontaneous pregnancy and the causes of infertility’, (2) ‘The course of IVF’, (3) ‘The different methods of fertilization (difference between insemination and micro-fertilization)’, and (6) ‘The schedule of IVF treatment’.

The explanations facilitated by a tablet terminal had a direct effect for these items. Tablet terminals have the major advantage in the ability to show videos or animations of pregnancy mechanisms, egg growth, or other in vivo processes. The ability to show novel things virtually may deepen the level of understanding.

On the other hand, when special treatment such as IVF is undertaken, the history of the treatment may become a problem. This particularly applies to bioethical matters, such as instances in which patients must choose a treatment after they receive limited facts (that are the most current knowledge) from a medical perspective. The timing of the embryo transfer in IVF is split between before and after the third day following fertilization. While the embryo transfer prior to the third day after fertilization is not physiologically advisable, this has a long history of being done during treatment. Therefore, more is known about the effects of this early embryo transfer on the children born as a result than about the embryo transfer from 3 days after fertilization. The safety of this practice is therefore not in question. The extended embryo transfer to five days after fertilization, meanwhile, has a short history and may cause problems in the future. Discrepancies were seen even within couples for answers to (4) ‘The culture period’. This is undoubtedly an item with which patients struggle quite a bit to understand and come to a decision. This issue is likely also reflected in the questionnaire. The level of understanding of this item is unlikely to change, regardless of whether it is ultimately presented in printed form or on a tablet terminal.

Next, item (7) ‘Things to take note of, such as consultation times’ is based on the assumption that many patients have jobs. In Japan, self-injections used for ovarian stimulation are limited to only a small range of products, and the Medical Care Act stipulates that the majority of preparations must be given at a medical institution. As a result, injections are proportional to the number of clinic visits, which are assumed to be the result of anxiety caused by the lack of balance between clinic consultation hours and work hours.

As for (5) ‘The treatment fees’, paper-based pamphlets specify all costs per treatment or procedure. The treatments and procedures used over the course of IVF therapy differ according to the cause of infertility, which means it is unclear which treatment or procedure should be performed prior to the start of therapy. Several patterns and examples are provided in the tablet terminal-based explanation. While this clearly reduces the number of patient queries, it is unlikely that patients will be 100% convinced about these money-related matters, regardless of the explanation method used.

The questionnaire in the remarks column had a question about ovarian stimulation, as well as a few questions about assisted hatching. Because we change the method of ovarian stimulation based on the ovarian function of the patients in this hospital, it might be hard to understand. We decided to adopt assisted hatching based on the thickness of the transparent zone of the egg. We believe that the meaning of thickness of the transparent zone is difficult for patients to understand, because patients believe that embryos collected at the same time are all in the same condition. However, all embryos actually have a different character.

This questionnaire survey clearly showed that a higher level of patient understanding and consent is achieved with tablet terminal-based IVF therapy explanations than with paper-based explanations. Moreover, shorter consultation times already have been reported [4].

Tablet terminal-based presentations designed to modify health-related behaviour during pregnancy are reported to be effective in both obstetric terms and in mental health care terms [5, 6].

At our hospital, we currently provide patients with introductory paper-based pamphlets in advance and undergo individual interviews at the hospital, before IVF therapy is begun. At present, this method compensates for any items that patients do not understand, because a sufficient supplementary explanation is provided.

Because the circumstances of each patient are different, the queries on which they focus also differ. The need for patient-tailored support will likely remain unchanged in the future.

The introduction of a tablet terminal explanation was able to shorten the consultation time by 2 h. Currently, we have changed the system from paper-based pamphlets to tablet terminals, which are taken home for the next day’s lesson before the consultation. We plan to evaluate patients’ understanding with this method in the future.

## Conclusion

The present study demonstrated that the spread of tablet terminal-based explanations leads to improved patient understanding, and that tablet terminals are an effective method to explain IVF. Because tablet terminals are effective tools for transmitting knowledge, they may also aid in explanations given to people from other countries through language translation, such as English [5–7].

## Abbreviations

IVF: In vitro fertilization
